# Hallux Sesamoid Nonunion: A Comprehensive Systematic Review of Current Evidence

**DOI:** 10.3390/jpm15080342

**Published:** 2025-08-01

**Authors:** Elena Artioli, Antonio Mazzotti, Gianmarco Di Paola, Federico Sgubbi, Gianmarco Gemini, Simone Ottavio Zielli, Cesare Faldini

**Affiliations:** 1 IRCCS Istituto Ortopedico Rizzoli, Via Giulio Cesare Pupilli 1, 40136 Bologna, Italygianmarco.dipaola@ior.it (G.D.P.); federico.sgubbi@ior.it (F.S.); gianmarco.gemini@ior.it (G.G.); simoneottavio.zielli@ior.it (S.O.Z.); cesare.faldini@ior.it (C.F.); 2 Department of Biomedical and Neuromotor Sciences (DIBINEM), Alma Mater Studiorum University of Bologna, 40123 Bologna, Italy

**Keywords:** sesamoid, nonunion, forefoot, foot, arthroscopy, bone graft

## Abstract

**Introduction:** The optimal management of hallux sesamoid fracture nonunions remains a subject of ongoing debate, particularly in the context of personalized medicine. This systematic review aimed to synthesize current evidence regarding surgical strategies for this rare but disabling condition. **Methods:** A comprehensive literature search was conducted in accordance with the PRISMA guidelines. **Results:** Six studies met the inclusion criteria, encompassing a total of 80 patients. Surgical techniques varied and included open and arthroscopic sesamoidectomy, autologous bone grafting (alone or combined with screw fixation), and percutaneous screw fixation. When reported, outcomes were generally favorable, with union rates ranging from 90.5% to 100% and with consistent postoperative improvements in clinical function. Complication and reoperation rates were both 6.5%. The most frequent reoperation was sesamoidectomy for persistent pain or nonunion, followed by hardware removal. **Conclusions:** Despite the limited and low-quality evidence, available data suggest that individualized surgical planning can lead to favorable outcomes with low complication rates. Sesamoidectomy remains the most reliable salvage procedure in refractory cases. These findings support a personalized, stepwise approach to treatment—prioritizing sesamoid preservation, when feasible, while reserving excision for symptomatic nonunions. Further studies are needed to validate tailored algorithms and refine patient-specific decision-making in this challenging clinical scenario.

## 1. Introduction

The sesamoid complex of the hallux, composed of the medial (tibial) and lateral (fibular) sesamoid bones embedded within the tendons of the flexor hallucis brevis, plays a critical role in the biomechanics of the first metatarsophalangeal joint [1]. The sesamoid bones enhance the mechanical advantage of the flexor hallucis brevis tendons, transmitting loads that can exceed 300% of body weight during the push-off phase of gait [2].

Despite their functional importance, the sesamoids are susceptible to a variety of disorders, including sesamoiditis, osteonecrosis, osteochondral fractures, chondromalacia, dislocations, and fractures [3,4]. Among these, fractures are relatively uncommon but clinically significant, especially in athletes and individuals engaged in high-impact activities. These injuries typically result from direct trauma, repetitive stress, or excessive cyclic weight-bearing loads [3,5].

Initial management of sesamoid fractures is usually conservative, involving rest, immobilization, and offloading strategies through orthosis [6,7,8,9]. While many fractures heal uneventfully, a subset may progress to nonunion, particularly when diagnosis is delayed [3,10]. Sesamoid nonunion can lead to persistent pain and limitations in activity, significantly affecting quality of life and athletic performance [8,11].

Various strategies, either conservative or surgical, have been described for the treatment of sesamoid nonunion. However, there is no consensus regarding the optimal approach, and evidence remains sparse and heterogeneous [3,9,12,13].

Given the rarity of sesamoid fracture nonunions and the lack of standardized treatment protocols, clinical decisions are often based on limited evidence and individual experience. In the context of personalized medicine, where treatment strategies should be tailored to patient-specific anatomical, functional, and lifestyle factors, a structured evaluation of current surgical options is essential. Therefore, this systematic review was conducted to analyze the available surgical techniques, clinical outcomes, and complication profiles associated with the management of sesamoid fracture nonunions. The aim is to provide an evidence-based overview that may support individualized clinical decision-making and guide future research toward the development of patient-centered treatment algorithms.

## 2. Materials and Methods

A comprehensive bibliographic search was performed in the Embase, PubMed, and Cochrane Library databases on 25 May 2025. The search strategy included combinations of the following keywords: “nonunion,” “hallux,” and “sesamoid fracture” through the string “nonunion” AND “hallux” AND “sesamoid fracture”. After removing duplicates, all retrieved records were screened by title and abstract to identify studies specifically focused on the treatment of nonunion in hallux sesamoid fractures. Subsequently, full-text versions of the potentially eligible articles were reviewed in detail. Only original research articles reporting objective outcome data on patients treated for sesamoid fracture nonunion were included in the final analysis.

The following exclusion criteria were applied:

1. Absence of postoperative objective outcome data;
2. Studies involving patients with various sesamoid disorders where specific data on nonunion were not distinguishable (mixed population);
3. Studies reporting fractures unhealed for less than nine months (the most commonly agreed-upon standard definition [14]) unless nonunion was explicitly stated;
4. Articles not published in English;
5. Cadaveric studies, case reports, technical notes, narrative reviews, editorials, or surgical technique articles lacking outcome data.

Study selection was conducted independently by two authors (GDP, EA). Discrepancies were resolved through discussion, with the senior author serving as the final arbiter when consensus could not be reached.

From the included studies, the following data were extracted:

- First author and year of publication;
- Study design and corresponding Level of Evidence (LOE);
- Number of patients and feet, mean age at time of surgery, sex distribution, and affected sesamoid;
- Type of surgical intervention, bone graft harvest site (if applicable), percentage of successful unions, mean time to radiographic union, and duration of follow-up;
- Preoperative and postoperative clinical outcomes;
- Complications and any subsequent reinterventions.

This review was conducted in accordance with the PRISMA (Preferred Reporting Items for Systematic Reviews and Meta-Analyses) guidelines. The methodological quality and risk of bias of non-randomized studies were assessed using the Newcastle–Ottawa Scale (NOS).

This review was not registered.

### Statistical Analysis

Continuous variables were reported as means with standard deviations or ranges, while categorical variables were presented as percentages. Data were collected and organized using Excel (Microsoft 365).

## 3. Results

### 3.1. Type of Studies

**Figure 1:**
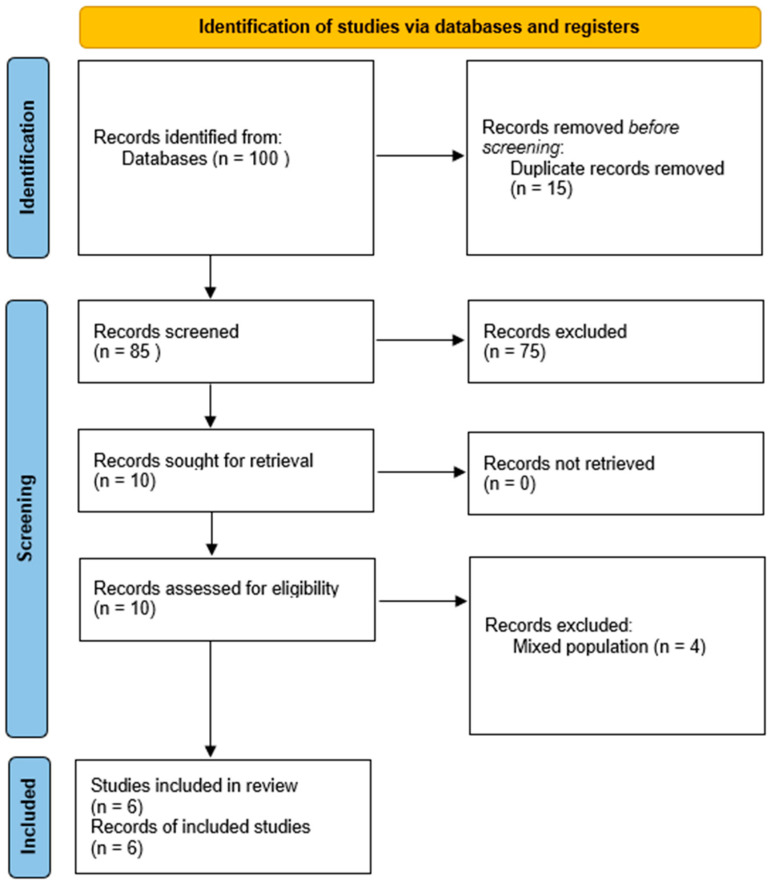
PRISMA flow diagram of the selection process.

The initial database search identified a total of 102 records—81 from Embase and 21 from PubMed. After the removal of duplicates, 85 unique articles remained. Title and abstract screening led to the exclusion of 75 studies that did not meet the inclusion criteria. The full texts of the remaining 10 articles were reviewed in detail, resulting in the inclusion of 6 studies in the final systematic analysis [11,15,16,17,18,19]. Four articles were excluded because they addressed various sesamoid disorders but specific data on nonunion were not distinguishable [20,21,22,23]. A detailed overview of the study selection process and reasons for exclusion is provided in the PRISMA flow diagram (Figure 1).

All studies included in the review were retrospective case series, corresponding to Level IV evidence. Risk of bias assessment, performed using the Newcastle–Ottawa Scale, indicated an overall low methodological quality. Specifically, three studies were classified as having a low risk of bias, while the remaining three were rated as high risk, as detailed in Table 1.

### 3.2. Population

The study population comprised 80 patients, each with a single affected foot, for a total of 80 treated feet. Of these, 37 were female (46.3%) and 29 male (36.3%). The mean age at the time of surgery was 30 years, with a range of 17 to 62 years. Regarding the anatomical distribution of nonunion, the tibial sesamoid was involved in 62 cases (77.5%), the fibular sesamoid in 17 cases (21.3%), and 1 case (1.3%) occurred in a congenital bipartite sesamoid (Table 2).

### 3.3. Surgical Procedures

The surgical techniques reported across the six included studies demonstrated considerable heterogeneity and personalization. Two studies adopted a resective strategy through sesamoidectomy (n = 29), while the remaining four pursued bone-preserving interventions aimed at achieving union through bone grafting and/or internal fixation (n = 51) (Table 2).

Sesamoidectomy was performed in two series. Bichara et al. treated 24 patients via open excision of the affected sesamoid [16], whereas Levaj et al. performed arthroscopic sesamoidectomy in 5 patients [17].

Bone-preserving procedures were reported in four studies, all of which attempted to promote healing of the nonunion through autologous bone grafting and/or internal fixation. Three of these studies employed autologous bone grafting (n = 42), performed either through an open [11] or arthroscopic approach [19], and in one case this was combined with open reduction and internal fixation using a screw [18].

Blundell et al. employed a minimally invasive approach, performing percutaneous fixation with a Barouk screw in nine patients without the use of a bone graft [15].

Among the three studies that incorporated bone grafting (n = 42), autologous bone was consistently used, though the donor site varied, including the first metatarsal [11], the iliac crest [19], and the calcaneus [18].

Postoperative management varies greatly with respect to immobilization, weight-bearing, use of orthoses, initiation of active and passive mobilization exercises, and recommendations for the gradual return to activities, as shown in Table 3.

### 3.4. Radiological and Clinical Results

Three studies reported the rate of fracture union, which was on average 96.8%, ranging from 90.5% to 100% [11,18,19]. Among the studies that provided data on time to union, the average duration for radiographic healing was between 2.9 and 3.0 months [11,18,19].

Clinical outcomes were assessed heterogeneously across studies and at varying follow-up intervals, with mean durations ranging from 6.0 to 56 months. The most commonly employed metric was the Visual Analogue Scale (VAS) for pain, reported in three studies. All demonstrated significant postoperative improvement, with preoperative scores averaging 65.75 and postoperative scores decreasing to a mean of 7.46 on a 0–100 scale [16,18,19].

Other clinical scoring systems varied among studies: Blundell et al. employed the American Orthopaedic Foot & Ankle Society (AOFAS) score, observing an improvement from 46.9 to 80.7 [15]. Nakajima et al. utilized the Japanese Society for Surgery of the Foot (JSSF) score, reporting an increase from 58.7 to 95.0 [19]. Park et al. used the Foot Function Index (FFI), which improved from 72.3 preoperatively to 8.2 postoperatively [18].

Return to sport or physical activity was documented in four studies, with a mean return time of approximately 5 months, ranging from 2 to 15 months [15,16,18,19] (Table 4).

### 3.5. Complications and Any Subsequent Reinterventions

The overall complication rate across the included studies was 6.25% (5 out of 80 patients). Anderson et al. reported three complications: two cases of persistent pain and tenderness attributed to unresolved nonunion, both subsequently managed surgically with sesamoidectomies, and one case of paresthesia along the course of the medial plantar digital nerve. Additionally, one reoperation for symptomatic bunion correction was performed in a patient with a preexisting deformity; notably, the hallux valgus angle and intermetatarsal angle remained stable from the time of the initial procedure [11].

Bichara et al. described one case of symptomatic hallux valgus deformity following medial sesamoid resection and one case of persistent pain; however, no reoperations were reported in this cohort [16]. Blundell et al. and Levaj et al. reported no postoperative complications, and no reinterventions were necessary in their respective series [15,17]. Finally, two studies reported no complications but documented reoperations. Nakajima et al. performed an arthroscopic sesamoidectomy in a patient with a symptomatic fibular sesamoid and a hypoplastic tibial sesamoid [19]. Park et al. reported two cases of hardware removal due to discomfort and patient anxiety [18] (Table 4).

## 4. Discussion

The present systematic review highlights the scarcity of high-quality evidence regarding the management of nonunion in hallux sesamoid fractures. The limited number of studies, coupled with small patient cohorts and generally low methodological quality, underscores the rarity of this clinical condition. To maintain the rigor of our analysis, the exclusion of case reports helped avoid further compromise of the overall quality of the review.

From the literature analyzed, it is evident that treatment can be individualized, and two principal surgical strategies emerge for the treatment of sesamoid nonunion: sesamoidectomy and sesamoid-preserving procedures.

Sesamoidectomy is an irreversible intervention that can be performed either via an open or arthroscopic approach [17]. Open techniques differ depending on whether the tibial or fibular sesamoid is involved. For the tibial sesamoid, a medial approach is commonly employed, with particular care taken to avoid injury to the medial plantar digital nerve, as postoperative paresthesia is a known complication [16]. For the fibular sesamoid, incisions are typically made either in the first interdigital space or through a direct plantar incision along the hallucal crease [11,16,18]. Notably, Bichara’s approach includes a diagnostic injection into the metatarsal–sesamoid joint to confirm pain relief prior to definitive excision, providing an elegant method to verify surgical indication in this irreversible procedure [16].

Sesamoid-preserving techniques reflect the standard principles for nonunion management, aiming to revitalize the fracture site through bone grafting and, where feasible, internal fixation [24]. In this context, fixation is sometimes omitted due to concerns about fracture displacement or hardware intolerance [25]. For instance, Park reported a hardware removal rate of 20% (2 out of 10 patients) within a two-year follow-up period due to discomfort [18].

An alternative surgical approach is reported by Moran et al. [21], whose article is not included in this review because it involves patients treated at 7 months after the fracture, while this review’s inclusion criteria set a limit of minimum 9 months according to the definition of nonunion [14]. Moran et al. do not directly address the sesamoids but instead utilize temporary surgical immobilization of the first metatarsophalangeal joint for eight weeks, employing either crossed wires or orthogonally placed two-hole plates. In a series of 32 patients, this method achieved a 94% union rate, with return to work at an average of 61 days and return to sport at 80 days postoperatively [21]. Although this study did not formally evaluate MTP joint motion before and after immobilization, such assessment would be valuable given the potential functional impact of prolonged joint immobilization.

Nonetheless, these outcomes are comparable to those reported in the included studies, which demonstrated union rates ranging from 90.5% to 100%, alongside significant improvements in clinical parameters, despite heterogeneity in outcome measures. Nakajima et al. noted that patients with congenitally distinct sesamoids and fracture tend to experience less favorable outcomes, highlighting the importance of anatomical considerations in prognosis [19]. Return to sport was a frequently reported outcome, with a mean time of 5.2 months (range 2–15). However, this parameter is undoubtedly influenced by both postoperative management and the age of the patient cohort. Regarding postoperative management, although several studies provided patients with guidelines on the timing of return to sport, the actual time required to resume sports activities was consistently longer [15,18]. Concerning age, it appears to be a more relevant factor: notably, the study by Park reported the longest time to return to sport (9.9 months), and it also involved the oldest patient cohort among all the included studies [18]. Therefore, the longer return-to-sport period observed in that study may be more closely related to the demographic characteristics of the population rather than to the surgical technique itself.

Moreover, complication rates associated with the surgical techniques were generally low. Although sesamoidectomy has been previously linked to postoperative complications [22,26], this review, which includes only two studies performing sesamoidectomy on 29 patients in total, did not reveal significant complication rates or require reoperations within those series. Interestingly, Bichara reported satisfactory outcomes in a cohort of 24 athletes undergoing sesamoidectomy, with a return to activity within approximately 11.6 weeks as already reported in the literature [27]. It is important to emphasize that sesamoidectomy is the most commonly reported reoperation for persistent pain or unresolved nonunion. This suggests that some authors advocate for a stepwise treatment algorithm, initially attempting sesamoid preservation and resorting to sesamoidectomy only after failure of reconstructive measures [11,19]. Such a personalized approach warrants further investigation and may serve as a foundation for developing a standardized treatment algorithm.

### Limitation

Limitations of this review include the small number of available studies, limited patient populations, and a generally high risk of bias. The inclusion criteria were very strict in order to reduce risk and select only the highest-quality articles. However, three studies were assessed as high risk of bias and differ from the others primarily due to their short follow-up periods [15,17,18]. In fact, two of them had a follow-up of just under two years, which is generally considered an acceptable threshold, while the study by Blundell reported only a 6-month follow-up [15]. No specific analytical methods, such as sensitivity analyses, were applied during the review to address the potential bias introduced by these high-risk studies. However, it should be noted that the absence of long-term complications or reoperations reported by the authors may be a consequence of the limited follow-up duration.

Furthermore, the marked heterogeneity in clinical outcome measures, with only the VAS being reported by more than one article, precluded more comprehensive statistical analyses. Nonetheless, this review represents the only systematic review of current evidence on this topic, providing a valuable overview of therapeutic options in a well-defined patient population with reliable results, as all included studies had a minimum follow-up exceeding three months—corresponding to the average time to bone union [11,18,19]. Unfortunately, it is not possible to confidently state that one surgical intervention is superior to another, as each treatment approach is supported only by individual case series. The only comparable parameters across the different interventions are pain scores and return to sport.

Therefore, no definitive conclusions can be drawn about the best treatment option. However, all the described procedures have yielded satisfactory outcomes. Future research should focus on larger, well-designed comparative studies with standardized outcome measures and longer follow-up periods to better determine the most effective treatment strategies for sesamoid nonunion.

## 5. Conclusions

Nonunion of hallux sesamoid fractures is an uncommon condition, with limited evidence available. The current literature is characterized by small case series of low methodological quality, and treatment strategies remain heterogeneous. Despite these limitations, two main surgical approaches can be identified: sesamoidectomy and sesamoid-preserving techniques involving bone grafting and, when feasible, internal fixation. Both strategies have demonstrated significant improvement in clinical scores in selected patients, with high union rates and good clinical outcomes. Complication rates appear low, although larger cohorts are lacking.

Sesamoidectomy remains the most commonly employed salvage option in cases of persistent symptoms or failed union. A patient-specific stepwise approach—initially aiming to preserve the sesamoid and reserving excision for refractory cases—warrants further investigation and may provide a basis for the development of a personalized treatment algorithm.

Given the rarity of this condition, further high-quality studies with standardized outcome reporting are needed to better define optimal treatment protocols. Moreover, a deeper understanding of post-traumatic sesamoid pathology and the pursuit of increasingly personalized treatment strategies are essential to improving patient outcomes and addressing the current gaps in evidence-based care.

## Figures and Tables

**Table 1:** Risk of bias assessment of the included studies through the Newcastle–Ottawa Scale.

	SELECTION				COMPARABILITY	OUTCOME			
**Authors and Years**	Representativeness of cases	Selection of controls	Ascertainment of exposure	Demonstration that outcome of interest was not present at start of study		Assessment of Outcome	Follow-Up Long Enough	Adequacy of Follow-Up	Total
*Anderson R.B. et al., 1997* [11]	-	-	★	★	-	★	★	★	**5** **★**
*Bichara D.A. et al., 2012* [16]	-	-	★	★	-	★	★	★	**5** **★**
*Blundell C.M. et al., 2002* [15]	-	-	★	★	-	★	-	★	**4** **★**
*Levaj I. et al., 2021* [17]	-	-	★	★	-	★	-	★	**4** **★**
*Nakajima K. et al., 2022* [19]	-	-	★	★	-	★	★	★	**5** **★**
*Park Y.H. et al., 2024* [18]	-	-	★	★	-	★	-	★	**4** **★**

**Table 2:** Demographic data of the selected articles.

Authors-Years	Study Design and LOE	Number of Feet (n. Patients)	Age at Treatment (Years)	Gender F:M	Affected Sesamoid	Surgical Procedure	Bone Graft Harvest Site
*Anderson R.B. et al., 1997* [11]	Retrospective case series (IV)	21 (21)	32.9	10F:11M	- 21 tibial	Open autologous bone grafting	Ipsilateral medial eminence of the first metatarsal head
*Bichara D.A. et al., 2012* [16]	Retrospective case series (IV)	24 (24)	32.2 ± 10.4 (17–54)	-	- 15 tibial - 9 fibular	Open sesamoidectomy	-
*Blundell C.M. et al., 2002* [15]	Retrospective case series (IV)	9 (9)	26 ± 7.0 (17–45)	3F:6M	- 5 tibial - 4 fibular	Percutaneous fixation with Barouk screw	-
*Levaj I. et al., 2021* [17]	Retrospective case series (IV)	5 (5)	21.2 ± 6.2 (14–39)	5F:0M	- 3 tibial - 2 fibular	Arthroscopic sesamoidectomy	-
*Nakajima K. et al., 2022* [19]	Retrospective case series (IV)	11 (11)	18.6 ± 10.3 (13–49)	3F:8M	- 9 tibial - 1 fibular - 1 distal part of congenital bipartite sesamoid	Arthroscopic autologous bone grafting	Iliac crest
*Park Y.H. et al., 2024* [18]	Retrospective case series (IV)	10 (10)	39.4 ± 14.0 (23–62)	6F:4M	- 9 tibial - 1 fibular	Open screw fixation and autologous bone grafting	Ipsilateral calcaneus
***Total***		80 (80)	30.0 (17–62)	37F:29M	- 62 tibial - 17 fibular - 1 congenital bipartite sesamoid		

**Table 3:** Postoperative management. PDO: postoperative day; MTP: metatarsophalangeal.

Authors and Years	Number of Feet (n. Patients)	Age at Treatment (Years)	Postoperative Management	Return to Sport/Activity (Months)
*Anderson R.B. et al., 1997* [11]	21 (21)	32.9	The patient was placed immediately into a short leg plaster splint. The patient remained non-weight-bearing for a period of 3 to 4 weeks, at which time a short leg walking cast was applied, again immobilizing the hallux. The cast was removed at 8 weeks. A soft, medial longitudinal arch support was prescribed and was used in conjunction with a firm-soled shoe. Active exercises were initiated, followed by gentle passive range of motion as symptoms permitted.	-
*Bichara D.A. et al., 2012* [16]	24 (24)	32.2 ± 10.4 (17–54)	The patients were instructed to begin weight-bearing as tolerated in a postoperative shoe with crutches for 7 to 10 days. Some patients used a removable walking boot for an additional 2 weeks.	2.9 ± 0.97 (2–6)
*Blundell C.M. et al., 2002* [15]	9 (9)	26 ± 7.0 (17–45)	Patients were mobilized with two crutches for one week, bearing weight as tolerated. They were then allowed to bear weight fully without aids. Running was allowed at six weeks with a gradual return to full activity at three months.	6.0
*Levaj I. et al., 2021* [17]	5 (5)	21.2 ± 6.2 (14–39)	All patients started with gentle passive range-of-motion exercises for the first MTP joint at PDO 3. At the end of the second postoperative week, the patients started with active and active-assisted ROM exercises for the first MTP joint. Strengthening and ROM exercises were initiated at the fourth postoperative week. For the first three postoperative weeks, the patients walked with the aid of two crutches and were allowed to bear weight as tolerated in a removable short-leg walking splint with a rocker sole. For the next six weeks, the patients were instructed to wear rocker bottom shoes. At that time, patients started to wear regular shoes. Patients returned progressively to sports three to six months after the surgery.	-
*Nakajima K. et al., 2022* [19]	11 (11)	18.6 ± 10.3 (13–49)	Passive plantar flexion of the hallux at PDO 1. Passive dorsiflexion and active plantar flexion were not allowed until 6 weeks after surgery. Walking with a postoperative shoe was started at PDO 1 until 6 weeks after surgery. Sport activities were initiated after confirmation of complete union at CT.	5.3 ± 3.6 (2–15)
*Park Y.H. et al., 2024* [18]	10 (10)	39.4 ± 14.0 (23–62)	A short leg splint was applied to immobilize the foot for 2 weeks, and patients were instructed on non-weight-bearing crutch walking. Subsequently, the splint was removed, and heel weight-bearing was allowed for 4 weeks. At 6 weeks postoperatively, the patients were transitioned to full weight-bearing and started physical therapy to improve the first metatarsophalangeal joint range of motion. Running was allowed at 8–10 weeks postoperatively, with a gradual return to full activity at 3 months.	9.9 ± 3.8
***Total***	80 (80)	30.0 (17–62)		5.2 (2–15)

**Table 4:** Summary of the outcomes in the selected articles.

Authors and Years	Achieved Union	Average Time Until Bone Union (Months)	Follow-Up (Months)	VAS	Clinical Scores	Return to Sport/Activity (Months)	Complications	Reoperations
*Anderson R.B. et al., 1997* [11]	90.5%	3.0	56 ± 27.2 (23–132)	-	-	-	- 2 persistent pain and tenderness (persistent nonunion) - 1 paresthesia along the course of the medial plantar digital nerve	- 2 sesamoidectomy (persistent nonunion) - 1 bunion correction (premorbid condition with HVA and IMA not progressed since the time of sesamoid grafting)
*Bichara D.A. et al., 2012* [16]	-	-	35 21 (8–70)	Pre-op. 62.0 ± 14.0, post-op. 7 ± 10	-	2.9 ± 0.97 (2–6)	- 1 patient developed a symptomatic hallux valgus deformity after the resection of the medial sesamoid - 1 persistent pain	No reoperations needed
*Blundell C.M. et al., 2002* [15]	-	-	6.0	-	- AOFAS: pre-op. 46.9 ± 9.75 (25–64) post-op. 80.7	6.0	No complications observed	No reoperations needed
*Levaj I. et al., 2021* [17]	-	-	20.6 ± 12.5 (4–54)	-		-	No complications observed	No reoperations needed
*Nakajima K. et al., 2022* [19]	100%	2.9 ± 0.8 (2–4)	38.4 ± 9.6 (25.2–57.6)	Pre-op. 72.1 ± 15.2 (50–100), post-op. 12 ± 26.7 (0–70)	- JSSF: pre-op. 58.7 ± 15.8 (42–87) post-op. 95.0 ± 11 (69–100)	5.3 ± 3.6 (2–15)	No complications observed	-1 arthroscopic sesamoidectomy (patient with affected fibular sesamoid and hypoplastic tibial sesamoid)
*Park Y.H. et al., 2024* [18]	100%	3.0	23.4 ± 14.5 (12–61)	Pre-op. 67.8 ± 13.5, post-op. 3.6 ± 4.8	- FFI: pre-op. 72.3 ± 8.7 post-op. 8.2 ± 8.3	9.9 ± 3.8	No complications observed	- 2 hardware removal due to discomfort and anxiety
***Total***	96.8%	2.95 (2–4)	35.4 (4–61)	Pre-op. 65.75, post-op. 7.46		5.2 (2–15)	6.25%	6.25%

## Data Availability

No new data were created or analyzed in this study. Data sharing is not applicable to this article.
